# Decreased intracellular IL-33 impairs endometrial receptivity in women with adenomyosis

**DOI:** 10.3389/fendo.2022.928024

**Published:** 2022-07-22

**Authors:** Bin He, Xiao-Ming Teng, Fan Hao, Mei Zhao, Zhi-Qin Chen, Kun-Ming Li, Qiang Yan

**Affiliations:** ^1^ Reproductive Medical Center, Shanghai First Maternity and Infant Hospital, Tongji University School of Medicine, Shanghai, China; ^2^ Center for Clinical Research and Translational Medicine, Yangpu Hospital, Tongji University School of Medicine, Shanghai, China

**Keywords:** adenomyosis, endometrial receptivity, IL-33, STAT3, HOXA10

## Abstract

Adenomyosis is a common benign uterine lesion that is associated with female infertility, reduced clinical pregnancy rate and high miscarriage risk. While it has been known that the impaired endometrial receptivity is implicated in infertility in patients with adenomyosis, the underlying mechanism remains unclear. In the present study, we showed that intracellular protein level of IL-33 was downregulated in the endometrium of patients with adenomyosis, and IL-33 expression status was shown to be positively correlated with that of HOXA10, an endometrial receptivity marker. The subsequent analysis indicated IL-33 overexpression led to the increase of HOXA10 expression and enhancement of embryo implantation *in vitro*, which was accompanied with induction of STAT3 phosphorylation. Meanwhile, cryptotanshinone, a potent STAT3 inhibitor, was found to significantly suppress the increase of HOXA10 expression and embryo implantation caused by IL-33 overexpression *in vitro*, revealing the critical role of STAT3 activity. Consistently, the positive relationship between IL33 and HOXA10 expression in the endometrium was verified in the analysis of adenomyosis mouse model.

## Introduction

Adenomyosis is a common benign uterine disorder that manifests as invasion of the endometrium in the muscular layer. According to epidemiological studies, many young women with pain, infertility, Abnormal Uterine bleeding (AUB) or even no symptoms are increasingly diagnosed with adenomyosis using imaging techniques such as transvaginal ultrasound or magnetic resonance (1). Adenomyosis is associated with a decreased clinical pregnancy rate, increased early miscarriage rate and infertility (2, 3). In our previous studies, we showed that impaired endometrial receptivity in patients with adenomyosis may be an important cause of infertility (4, 5). However, the specific mechanism of decreased endometrial receptivity is still unclear.

The research of endometrial receptivity was carried out about 80 years ago, followed with the technique of embryo transplantation (6, 7). Because not all high-quality embryo transfers achieve successful implantation, Navot et al. proposed a “window of implantation” to further describe endometrial receptivity, which is considered to be a rather short and important endometrial phase (7). Many studies have focused on the measurement of endometrial receptivity, including histologic criteria, electronic observation, and biochemical markers. The evaluation methods used tend to be highly microscopic and specific, and many biochemical markers have been discovered (8). The key transcription factor homeobox A10 (HOXA10) is considered to be a biomarker for the window of embryo implantation (9). HOXA10 has a conserved homeobox sequence that specifically recognizes the TTAT or TAAT sequence in the promoter region of the downstream gene. It can also activates or inhibits the binding of β3 integrin (ITGB3) (9), metalloproteinase MMP26 (10), and FK506 Protein 4 (FKBP4) (11, 12) as well as the expression of target genes such as the homeobox structural gene EMX2 (13), which has been demonstrated to regulate endometrial receptivity and embryo adhesion. The expression of HOXA10 in the endometrium changes with the menstrual cycle, and the highest expression level appears during the mid-secretory phase, which is the “implantation window period”. Thus, the downregulation of HOXA10 in the endometrium is considered the main reason for impaired endometrial receptivity in some women, such as those with endometriosis (10), hydrosalpinx (11), and repeated implantation failure (12). Previous studies also found that HOXA10 expression was downregulated in the endometria of patients with adenomyosis, which led to impaired endometrial receptivity (13). However, the mechanism of abnormal HOXA10 expression is unclear.

Interleukin-33 (IL33), a novel member of the IL1 family, was discovered to be a nuclear factor in the high endothelial vein (NF-HEV) in 2003 (14). In 2005, IL33 was determined to be as a specific extracellular ligand for ST2, which belongs to the orphan IL-1 receptor family. Since its discovery, IL33 has been found to be expressed widely in tissues and is mainly located in the nucleus. Although some studies have suggested that IL33 acts as a transcriptional repressor, the function of intracellular IL33 needs to be further clarified (15). When facing trauma or infection, IL33 is released outside of cells as an endogenous danger signal or an alarm element. IL33 has been reported to play a major role in many diseases, including asthma, anaphylactic shock, cardiovascular disease, and rheumatoid arthritis. However, there has been little research on the role of IL33 in reproduction. A previous study found that the IL33/ST2 pathway may participate in the pathogenesis of endometriosis and that IL33 serum cytokine levels in women with adenomyosis were decreased compared to those in controls (16, 17). However, the function of IL33 in the endometrium is unknown.

Previous evidence underlies the important role of IL33/ST2 as a critical cytokine in pregnancy (18). Nevertheless, the functional roles of intracellular IL33 in the endometrium during the progression of embryo implantation have not been elucidated. Combined with previous evidence, we hypothesized that the intracellular IL33 in the eutopic endometrium of the infertile women with adenomyosis may cause failure of embryo implantation. The purpose of this study is to confirm the function of intracellular IL33 on embryo implantation and further clarify the mechanism of infertility in adenomyosis.

## Materials and methods

### Patient sample collection

20 infertile patients with adenomyosis and 20 fertile controls participated in this study. The endometrium samples were obtained during the 20-22 day of the menstrual cycle, which is the secretory phase. This study was approved by the Ethics Committee of Shanghai First Maternity and Infant Health Hospital. In this study, patients who experienced at least one normal pregnancy or delivery were included in the fertile control group. The diagnostic criteria for adenomyosis were described in our previous study (13). All participants had regular menstrual cycles of approximately 28-31 days, and none of the patients received hormone therapy within three months prior to sampling. Patients with PCOS, endometrial polyps, hydrosalpinx or uterine fibroids were excluded. Patient information is summarized in **Table 1**.

**Table 1:** Patients information.

	Nor (n=20)	Ade (n=20)	P
**Age (years)**	**29.3±0.6**	**28.8±0.7**	**ns**
**Body mass index (kg/m^2^)**	**21.4±0.5**	**21.8±0.4**	**ns**
**Smoker**	**6 (30.0)**	**4 (20.0)**	**ns**
**Drinker**	**7 (35.0)**	**8 (40.0)**	**ns**
**Menstrual cycle (days)**	**29.6±0.8**	**29.9±0.3**	**ns**
**Infertility duration (years)**	**3.3±2.3**	**3.3±1.4**	**ns**
**Basal sex hormone**			
** FSH (IU/L)**	**6.78±1.39**	**6.77±1.57**	**ns**
** LH (IU/L)**	**5.29±3.57**	**5.21±1.55**	**ns**
** E_2_ (pg/ml)**	**40.92±9.77**	**44.51±9.64**	**ns**
** P (ng/ml)**	**0.53±0.15**	**0.58±0.21**	**ns**
** T (ng/ml)**	**0.41±0.19**	**0.42±0.19**	**ns**

The data are presented as the mean ± SD unless otherwise indicated. Nor, Normal; Ade, Adenomyosis; ns, no significant. P < 0.05 was considered significant.

### Cell culture, steroid hormones and inhibitors

Ishikawa cells and BeWo cells were cultured in DMEM supplemented with 10% (v/v) foetal bovine serum (Gibco BRL/Invitrogen, Carlsbad, CA, USA) and 1% penicillin/streptomycin (HyClone Laboratories, South Logan, UT, USA) at 37 °C in an atmosphere of 5% CO_2_/95% air. Ishikawa cells were transfected with overexpression plasmids or siRNA. The administration of 17β-oestradiol (E, 10 ^−8^ M) and progesterone (P, 10 ^−6^ M) for different times was conducted in Ishikawa cells as described in the figure legends. STAT3-dependent signalling pathway inhibitor (Cryptotanshinone, 4.6 µM) (SigmaAldrich, St Louis, MO, USA) was added to the culture medium before the plasmids were transfected into Ishikawa cells.

### RNA isolation and quantitative real-time PCR

Total RNA from Ishikawa cells, human endometrial tissues or mouse uteri was extracted with TRIzol reagent (Life Technologies, NY, USA). According to the manufacturer’s instructions, purified total RNA (1 μg) was reverse-transcribed into cDNA using the PrimeScript RT reagent kit (Bio–Rad, Hercules, CA, USA). Quantitative real-time PCR was performed using a MyiQ Single-Colour Real-Time PCR Detection System (BIO-RAD Laboratories, Hercules, CA, USA). The IL33, HOXA10 and ST2 mRNA expression levels were normalized to GAPDH with the 2^–△△CT^ method. The primer sequences for the indicated genes are shown in **Table 2**.

**Table 2:** Primer Sequences for qPCR.

Genes	Sequence of primers 5’ to 3’	
**IL33**	**Forward**	**GTGACGGTGTTGATGGTAAGAT**
	**Reverse**	**AGCTCCACAGAGTGTTCCTTG**
**ST2**	**Forward**	**ATGGGGTTTTGGATCTTAGCAAT**
	**Reverse**	**CACGGTGTAACTAGGTTTTCCTT**
**HOXA10**	**Forward**	**CTCGCCCATAGACCTGTGG**
	**Reverse**	**GTTCTGCGCGAAAGAGCAC**
**GAPDH**	**Forward**	**TGTGGGCATCAATGGATTTGG**
	**Reverse**	**ACACCATGTATTCCGGGTCAAT**

### Western blot assay

Proteins are extracted from Ishikawa cells, human endometrial tissues or mouse uteri. Through the Bradford assay (Bio–Rad Laboratories), the protein content was measured, and equal amounts (20 μg) of protein were separated by SDS–PAGE. Immunoblotting was performed with primary antibodies against IL33 (Proteintech, USA, 1:1000), HOXA10 (Cell Signaling Technology, USA, 1:1000), ITGB3 (Cell Signaling Technology, USA, 1:1000), STAT3 (Cell Signaling Technology, USA, 1:1000), p-STAT3 (Cell Signaling Technology, USA, 1:1000) or GAPDH (Bioworld Technology, USA, 1:10000), followed by incubation with a horseradish peroxidase (HRP)-conjugated secondary antibody. An enhanced chemiluminescence kit (Amersham Biosciences Corp., USA) was used to detect the bands. Finally, the densitometric analysis of each band was performed with Quantity-one (Bio–Rad Laboratories) software.

### Attachment assay of BeWo spheroids to Ishikawa cells

Ishikawa cells were digested and inoculated into a 24-well culture plate. When the cells reach 80%, the indicated plasmids were transfected into the cells: Mix the plasmids to be transfected with Lip2000 reagent (Invitrogen, China) in Opti-MEM^®^ Reduced Serum Medium at a ratio of 1:2. 8h after transfection, the serum-free medium was replaced with normal medium containing 10% serum. After 48 h, 50 BeWo cell spheroids (at the same diameter of 150-200 μm) were transferred to a confluent monolayer of Ishikawa cells in each group. After incubation for 2 h at 37 °C, each well of the culture plate was washed with PBS 3 times, and the unattached spheroids were washed away. Spheroids were fixed in 4% paraformaldehyde at room temperature for 30 min, and the adhesion state of BeWo spheroids was observed and counted under a stereoscopic dissecting microscope, which was used to calculate the adhesion efficiency.

### Transfection and luciferase assays

Ishikawa cells were digested and seeded into cell culture plates. Upon reaching 80% confluence, the cells were transfected with the indicated plasmids. After culturing for 48 h, 200 ng of the pGL3-ITGB3-Luc reporter gene plasmid was transiently transfected into Ishikawa cells using Lipofectamine 2000 transfection reagent. After incubating for 24 h, the cells were lysed with reporter gene lysis solution according to the method described in the Dual-Luciferase Reporter Assay Kit, and the supernatant was collected to measure the luciferase activity according to the Promega (Madison, WI, USA) Dual Luciferase Reporter Assay System.

### Adenomyosis mouse model

Male and female ICR mice were mated in a 1:1 ratio. On the 2nd-5th day after birth, the newborn mice were separated from the mother mice every morning. After starvation for 5-6 hours, the adenomyosis group was drip-fed 5 µl/g (weight) of tamoxifen mixed in a peanut oil/lecithin/condensed milk mixture, and the control group was drip-fed only the peanut oil/lecithin/condensed milk mixture. After drip feeding, the mice were placed back in the same cage with the mother mice. All mice were exposed to light for 12 h every day and were fed by female mice from 1 to 21 days of age. From 22 days onwards, they were separated from the female mice. The mice were sacrificed at six months old. HE staining and immunohistochemistry were used to verify whether the model was successfully constructed.

### Immunohistochemistry

Human endometrial tissue or mouse uterine sections (5 μm) were deparaffinized in xylene and ethanol. Incubation with 3% H_2_O_2_ removed endogenous peroxidase. Slides were blocked with 1.5% normal rabbit blocking serum at room temperature for 45 min. Then, the sections were incubated at 4 °C with primary anti-IL33 antibody (1:200; Affinity Biosciences, China) or anti-HOXA10 antibody (1:50; Abcam, USA). After washing with PBS, the sections were incubated with sufficient peroxidase label edpolymer secondary antibody for 30 min at room temperature. Finally, the sections were incubated with enough DAB for 2-5 min at room temperature until a brown colour developed. The slides were washed with PBS, and the staining was observed with a microscope. Nonspecific rabbit IgG and goat IgG was used as a negative control, and nonspecific staining was not detected in the controls.

### HE staining

Mouse uterus section slides (5 μm) were deparaffinized in xylene and ethanol. The tissue samples were soaked and washed in PBS solution three times for 5 min each. Then, the slides were stained with haematoxylin solution. After staining, the excess haematoxylin staining solution was washed with distilled water. 1% hydrochloric acid ethanol was used for differentiation. After differentiation, the tissue sections were rinsed with double distilled water, and a weakly basic blue-stimulating solution was added to the tissue sections to stain the nuclei blue. After washing with double distilled water, tissue sample sections were stained with eosin for 3 minutes. After staining, the tissue sections were dehydrated in a gradient manner. The dehydrated tissue sample sections were soaked in xylene twice for 4 min each time, and then the tissue samples were dried and sealed with neutral gum. Finally, the sections were observed and imaged under a microscope.

### Statistical analysis

Each result is shown as the mean ± SD of three independent experiments. Two-tailed Student’s t-test was used to compare the mean expression values of two treatment groups; One-way ANOVA was performed for comparisons among more than two groups. Pearson’s correlation analysis was used to assess the relationship between IL33 and HOXA10. P-values < 0.05 were considered statistically significant.

## Results

### IL33 is aberrantly expressed in the endometria of women with adenomyosis

**Figure 1:**
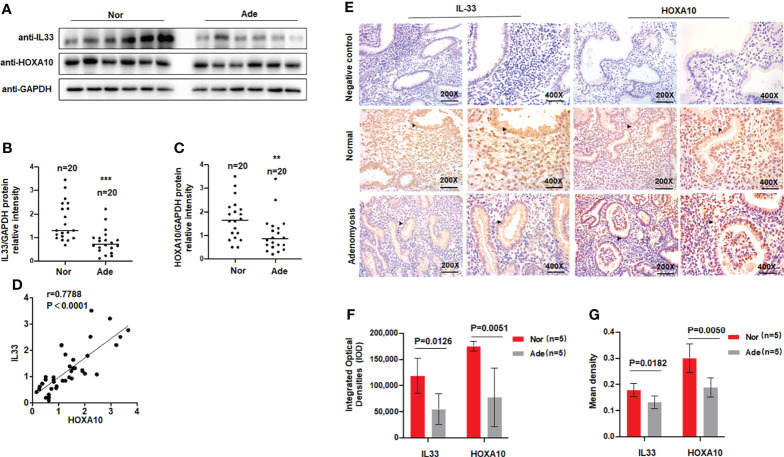
IL33 is aberrantly expressed in the endometria of women with adenomyosis. **(A)** IL33 and HOXA10 protein expression in normal (n = 20) and patients with adenomyosis (n = 20) was analyzed by Western blot. **(B)** The intensities of IL33 signals were quantified from the 20 samples and normalized. to GAPDH. ***p < 0.001. **(C)** The intensities of HOXA10 signals were quantified from the 20 samples and. normalized to GAPDH. **p < 0.01. **(D)** Correlation between IL33 and HOXA10 protein expression levels (r = 0.7788, p < 0.0001). **(E)** Timed mid-secretory endometrial biopsies from healthy control and infertile women with adenomyosis were analyzed using immunohistochemistry (IHC). Rabbit or Goat IgG was used as the negative control. The arrows show the decreased IL33 and HOXA10 conjugates in the endometrial epithelium cell. **(F)** The integrated optical densities (IOD) of the expression of IL33 and HOXA10 in the endometrium using Image-Pro Plus System 6.0 image analysis software. **(G)** The mean density of the expression of IL33 and HOXA10 in the endometrium. using Image-Pro Plus System 6.0 image analysis software.

A previous study found that serum levels of the cytokine IL33 were decreased in patients with adenomyosis (17), so we examined the levels of IL33 in the endometria of patients with adenomyosis. Patient information is shown in **Table 1**. We extracted total protein from endometrial tissues and detected IL33 protein expression *via* Western blotting. The results showed that the protein level of IL33 was significantly downregulated in the endometrial tissue of patients with adenomyosis (n=20) compared with the fertile controls (n=20) (**Figures 1A, B**). The protein level of HOXA10 was also significantly decreased (**Figures 1A, C**). In addition, the results indicated that the protein levels of IL33 and HOXA10 were positively related (**Figure 1D**). We further detected the expression and localization of IL33 in the endometrium by immunohistochemical staining (**Figure 1E**). We found that in the endometria of patients with adenomyosis, both IL33 and HOXA10 expression were lower. The results showed that IL33 was mainly localized in the nucleus of epithelial cells, similar to the fertile controls. In addition, we quantified the expression of IL33 and HOXA10 in the endometrium using Image-Pro Plus System 6.0 image analysis software. The integrated optical densities (IOD) of IL33 for the normal controls (n=5) was 118,261 ± 33,491, while that for the patients with adenomyosis (n=5) was 54,415 ± 29,457 (p=0.0126); the IOD of HOXA10 for the normal controls (17,5024 ± 9,531) was significantly higher than that of the patients with adenomyosis (77,558 ± 56,198), p=0.0051 (**Figure 1F**). In addition, the mean expression of IL33 in normal controls (0.1783 ± 0.025) was higher than that in adenomyosis (0.1415 ± 0.0071), p=0.00182; the mean densities of HOXA10 in the fertile and adenomyosis individuals were 0.3003 ± 0.055 and 0.1878 ± 0.036, p=0.0050 (**Figure 1G**).

### IL33 could induce HOXA10 expression

**Figure 2:**
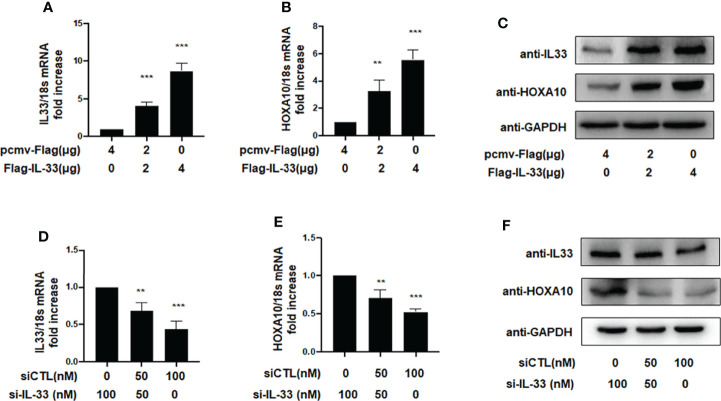
IL33 could induce HOXA10 expression. **(A)** Ishikawa cells were transfected with overexpression plasmid Flag-IL33 or pcmv-Flag negative control. IL33 mRNA levels were measured by qRT-PCR. ***p < 0.001. **(B)** Ishikawa cells were transfected with overexpression plasmid Flag-IL33 or pcmv-Flag negative control. HOXA10 mRNA levels were measured by qRT-PCR. **p < 0.01, ***p < 0.001. **(C)** Ishikawa cells were transfected with overexpression plasmid Flag-IL33 or pcmv-Flag negative control. IL33 and HOXA10 protein levels were measured by Western blot. **(D)** Ishikawa cells were transfected with si-IL33 or negative control. IL33 mRNA levels. were measured by qRT-PCR. **p < 0.01, ***p < 0.001. **(E)** Ishikawa cells were transfected with si-IL33 or negative control. HOXA10 mRNA levels were measured by qRT-PCR. **p < 0.01, ***p < 0.001. **(F)** Ishikawa cells were transfected with si-IL33 or negative control. IL33 and HOXA10 protein levels were measured by Western blot.

Previous studies demonstrated that the downregulation of HOXA10 in the endometrium was related to impaired endometrial receptivity in patients with adenomyosis (13). However, the molecular mechanism of HOXA10 downregulation in adenomyosis patients is unclear. In the present study, we utilized liposomes to transfect the high-expression plasmid Flag-IL33 into IK cells and verified the overexpression of IL33 by qRT–PCR and Western blot assays (**Figures 2A, B**). The results showed that overexpression of IL33 could significantly promote the mRNA and protein expression of HOXA10 (**Figures 2B, C**). When IL33 was silenced (**Figure 2D**), the HOXA10 mRNA and protein levels were reduced (**Figures 2E, F**).

### IL33 participates in embryo implantation

**Figure 3:**
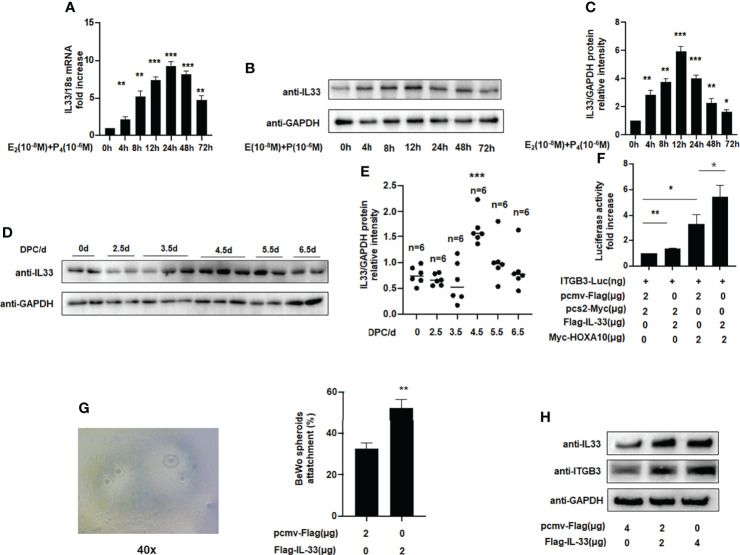
IL33 participates in embryo implantation. **(A)** Ishikawa cells were administered estrogen (10 ^–8^ M) and progesterone (10 ^–6^ M) at different times, as indicated. IL33 mRNA levels were measured by qRT-PCR. **p < 0.01, ***p < 0.001. **(B)** Ishikawa cells were administered estrogen (10 ^–8^ M) and progesterone (10 ^–6^ M) at different times, as indicated. Whole-cell lysates were analyzed by western blot analysis with the indicated antibodies. **(C)** Protein levels were normalized to GAPDH protein expression level in **(B)** (**p< 0.01, ***p < 0.001. vs control). **(D)** Western blot analysis of uterine IL33 during the peri-implantation period in mice, with the strongest decreased signal detected at 4.5 dpc. **(E)** Protein levels were normalized to GAPDH protein expression level in **(D)** (**p< 0.01, ***p < 0.001. vs control, n is shown in each group). **(F)** Ishikawa cells were transfected with ITGB3-Luc, Myc-HOXA10 (2 μg), and Flag-IL33 (2 μg), as indicated. After 48 h, the luciferase activities were measured and are presented as fold induction. Values represent the mean ± SEM (n = 3), *p < 0.05, **p < 0.01. **(G)** BeWo spheroids (150–200 μm diameter) were attached to Ishikawa cells after 2 h of co-culture. Adhesion experiments with BeWo spheroids attached to the Ishikawa cell monolayer. The data are the average of three independent experiments. An ANOVA test was used to compare the percentage of the attached spheroids with each treatment in comparison to the control. The error bars indicate SD of three independent experiments. Values represent the mean ± SEM (n = 3), **p < 0.01. **(H)** Ishikawa cells were transfected with Flag-IL33 (2 μg) as indicated. Whole-cell lysates were analyzed by western blot analysis with the indicated antibodies.

**Figures 3A–C** shows that endogenous IL33 mRNA and protein levels were increased in Ishikawa cells treated with oestrogen and progesterone in a time-dependent manner. To determine whether IL33 participates in regulating embryo implantation, we found that IL33 expression was dramatically increased 4.5 days post-coitus (dpc) in pregnant mice (**Figures 3D, E**) (p < 0.01). In the luciferase reporter assay, we found that ITGB3 activity, which is transcriptionally regulated by HOXA10, was enhanced in IL33-overexpressing IK cells (**Figure 3F**). In addition, the BeWo spheroid attachment assay confirmed that IL33 expression promoted the adhesion of BeWo cell spheroids to Ishikawa cells (**Figure 3G**). Moreover, enhanced IL33 expression induced ITGB3 protein levels (**Figure 3H**).

### ST2 silence dose not affect the IL33-promoted HOXA10 expression and embryo implantation *in vitro*

**Figure 4:**
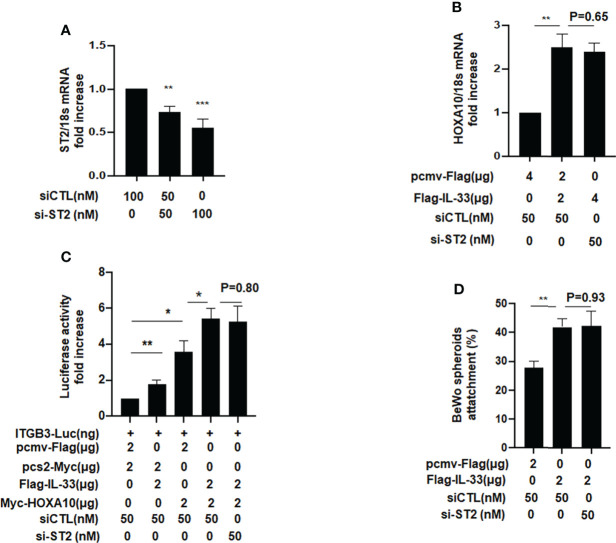
ST2 silence dose not affect the IL33-promoted HOXA10 expression and embryo implantation *in vitro.* **(A)** Ishikawa cells were transfected with si-ST2 or negative control. ST2 mRNA levels were measured by qRT-PCR ** p < 0.01, *** p < 0.001. **(B)** Ishikawa cells were transfected with si-ST2 or negative control. After 24h, IL33. was overexpression *via* the transfection of Flag-IL33. HOXA10 mRNA levels were measured by qRT-PCR. ** p < 0.01 **(C)** Ishikawa cells were transfected with si-ST2 or negative control. After 24h, ITGB3-Luc, Myc-HOXA10 (2 μg), and Flag-IL33 (2 μg) were transfected into the cells, as indicated. After 48 h, the luciferase activities were measured and are presented as fold induction. Values represent the mean ± SEM (n = 3), *p < 0.05, **p < 0.01. **(D)** BeWo spheroids (150–200 μm diameter) were attached to Ishikawa cells after 2 h. of co-culture. Adhesion experiments with BeWo spheroids attached to the Ishikawa cell monolayer. The data are the average of three independent experiments. An ANOVA test was used to compare the percentage of the attached spheroids with each treatment in comparison to the control. The error bars indicate SD of three independent experiments. Values represent the mean ± SEM (n = 3), **p < 0.01.

When IL33 acts as a cytokine, it binds to the ligand ST2 on the surface of the cell membrane, thereby causing a series of signal changes (14). Meanwhile, some studies have also shown that intracellular IL33 may play a role in regulating the expression of transcription factors (14). To further study the underlying mechanism, we silenced ST2 when IL33 was overexpressed and found that the promotive effects of IL33 on HOXA10 was not affected (**Figures 4A, B**). In the luciferase reporter assay, we found that the downregulation of ST2 expression does not affect the IL33-enhanced transcriptional activity of HOXA10 and the IL33-improved BeWo spheroids adhesion (**Figures 4C, D**). These results suggested that intracellular IL33 may play a role independently of ST2.

### IL-33 enhances embryo implantation *via* phosphorylation of STAT3

**Figure 5:**
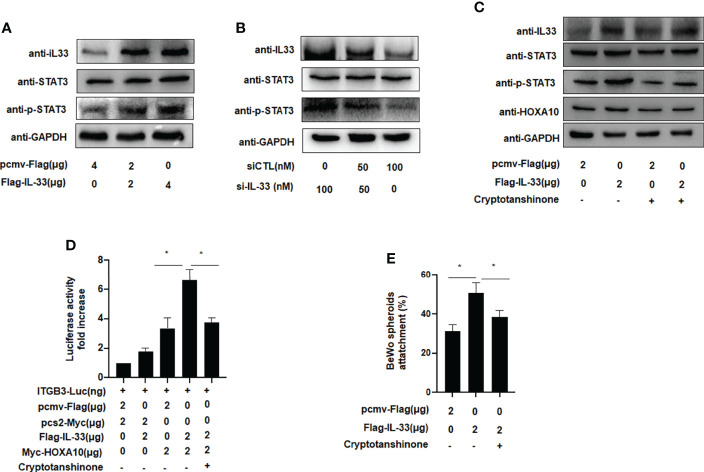
IL-33 enhances embryo implantation *via* phosphorylation of STAT3. **(A)** Ishikawa cells were transfected with Flag-IL33 (2 μg). IL33, STAT3 and p-STAT3 protein levels were analyzed by western blot analysis. **(B)** Ishikawa cells were transfected with siIL33 (50 or 100 nM), Whole-cell lysates. were analyzed by western blot analysis with the indicated antibodies. **(C)** Ishikawa cells were administrated with cryptotanshinone (4.6 μM) for 8h before. the transfection of Flag-IL33 (2 μg), Whole-cell lysates were analyzed by western blot analysis with the indicated antibodies. **(D)** Ishikawa cells were administrated with cryptotanshinone (4.6 μM) for 8h before. the transfection of ITGB3-Luc, Myc-HOXA10 (2 μg), and Flag-IL33 (2 μg), as indicated. The luciferase activities were measured and are presented as fold induction. Values represent the mean ± SEM (n = 3), *p < 0.05. **(E)** Ishikawa cells were administrated with cryptotanshinone (4.6 μM) for 8h before the. transfection of Flag-IL33 (2 μg). BeWo spheroids (150–200 μm diameter) were attached to Ishikawa cells after 2 h of co-culture. Adhesion experiments with BeWo spheroids attached to the Ishikawa cell monolayer. The data are the average of three independent experiments. An ANOVA test was used to compare the percentage of the attached spheroids with each treatment in comparison to the control. The error bars indicate SD of three independent experiments. Values represent the mean ± SEM (n = 3), *p < 0.05.

We further explored the molecular mechanism by which IL33 promoted embryo adhesion, and we found that phosphorylation of STAT3 was enhanced after IL33 was overexpressed in IK cells (**Figure 5A**). When IL33 expression was silenced, STAT3 phosphorylation was subsequently attenuated (**Figure 5B**). After treatment with cryptotanshinone (4.6 µM), an inhibitor of p-STAT3, the promotion of HOXA10 by IL33 was weakened (**Figure 5C**). Furthermore, when the phosphorylation of STAT3 was blocked by cryptotanshinone, the activity of ITGB3 and the adhesion of BeWo cell spheroids were also decreased (**Figures 5D, E**). These results demonstrated that IL33 increased endometrial receptivity *via* phosphorylation of STAT3.

### IL33 expression was decreased in a mouse model of adenomyosis

**Figure 6:**
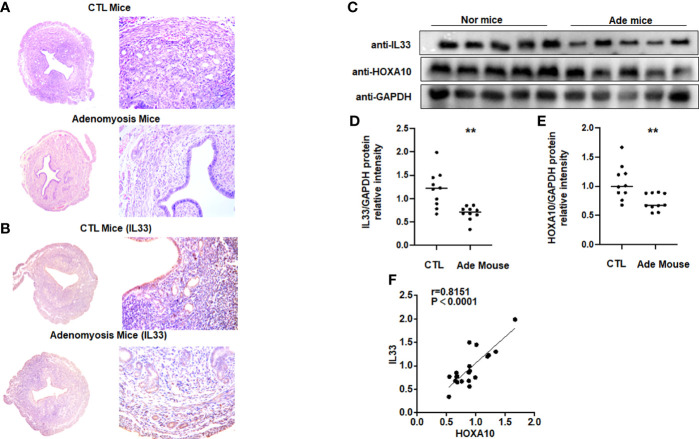
IL33 expression is decreased in a mouse model of adenomyosis. **(A)** HE staining confirmed that the mouse model of adenomyosis was successfully constructed. **(B)** Uterus from control mouse and adenomyosis mouse model were analyzed using immunohistochemistry (IHC). Rabbit IgG was used as the negative control. The arrows show the decreased IL33 conjugates in the endometrial cell epithelium. **(C)** IL33 and HOXA10 protein expression in control (n =10) and mouse with adenomyosis (n=10) was analyzed by Western blot. **(D)** The intensities of IL33 signals were quantified from the 10 samples and normalized to GAPDH. **p < 0.01. **(E)** The intensities of HOXA10 signals were quantified from the 10 samples and normalized to GAPDH. **p < 0.01. **(F)** Correlation between IL33 and HOXA10 protein expression levels (r = 0.8151, p < 0.0001).

We utilized HE staining to ensure that we successfully constructed a mouse model of adenomyosis. HE staining showed that endometrial glands appeared in the myometrium of the mouse uterus (**Figure 6A**). **Figure 6B** showed that IL33 mainly localized in the epithelial cells of the mouse uterus and decreased in the adenomyosis mouse model. We further found that IL33 protein expression, consistent with the HOXA10 level, was dramatically downregulated compared with that in the control group (**Figures 6C–E**). In addition, IL33 and HOXA10 protein expression was positively related (**Figure 6F**).

## Discussion

Adenomyosis has a negative impact on fertility, accounting for 25% of infertile women. Among women who underwent IVF therapy, adenomyosis deceased the clinical outcome (19–21).However, the underlying mechanism which adenomyosis cause implantation failure remains unclear. This study first demonstrated that the reduced IL33 expression in the endometrium impaired embryo implantation through modulation of HOXA10 expression.

In 1840, pathologists Carl von Rokitansky first described adenomyosis (22). Adenomyosis is believed to be a benign gynaecological disease in which endometrial glands and stroma are present in the myometrium. With the continuous development of diagnostic criteria, the incidence of adenomyosis is increasing, especially in reproductive women (23).Understanding how adenomyosis leads to infertility is complex; some studies have shown that, physically speaking, adenomyosis affects uterine motility and hinders sperm transport (24), while others have suggested that adenomyosis impairs decidualization and embryo implantation by impacting molecular expression (4, 25). The endometrial receptivity marker HOXA10 and its downstream target molecule ITGB3 were both decreased in the adenomyosis mouse model as well as during the secretory phase in patients with adenomyosis (26–28). However, the underlying mechanism remains unclear.

Changes in immune cells and the imbalance between proinflammatory and anti-inflammatory cytokines are involved in the subfertility of women affected by adenomyosis (29). Some studies have focused on macrophages and T cells, which were found to be increased in the endometrium from adenomyosis-affected uteri compared to disease-free uteri (30, 31). These changes ultimately led to the abnormal secretion of cytokines and other immune factors. Proinflammatory factors such as IL1β, IL6, TNFα and NF-kB and anti-inflammatory factors such as IL10, TGF-β1, HGF, and EGF were also found to be dysregulated (29). This dysregulation modifies the immune environment and causes the abnormal expression of some proteins related to embryo implantation. Few studies have focused on the effect of IL33 on adenomyosis. Only one study investigated the serum IL33 levels in adenomyosis-affected women (17). In the study, the researcher analysed the serum cytokine profiles in different types of adenomyosis: diffuse adenomyosis (DIF-ADE), focal adenomyosis (FOC-ADE), and combination of focal and diffuse lesions (FOC/DIF-ADE) (17). They demonstrated that the serum IL33 levels were reduced in the adenomyosis, especially in the type of FOC/DIF-ADE. In our study, we illustrated that in both the endometrium of infertile women and the mouse model with adenomyosis, IL33 expression was downregulated. However, we did not undergo further studies on the different types of adenomyosis. Previous studies have proven the lower embryo implantation rate and higher miscarriage rate in adenomyosis-affected women undergoing IVF (32). Some researchers proposed that the Uterine Junctional Zone (JZ) thickness evaluated by MRI is the best predictive factor of implantation failure (33). When the average JZ thickness was more than 7 mm, the implantation failure was found to be higher (34). According to the classification of adenomyosis *via* MRI examination, the JZmax in DIF-ADE should be more than 12mm, which would be a major factor affecting embryo implantation (35, 36). In our study, we found endometrial IL33 expression was positively correlated to HOXA10 expression and may involve embryo implantation in patients with adenomyosis. Regarding to the embryo implantation, we supposed that the expression of IL33 in the endometrium should be more affected in the type of DIF-ADE. This proposal requires further research, and studies on deeper molecular mechanism should be conducted in the future.

IL33 is widely expressed in many tissues and predominantly located in tissue cell types, including endothelial cells, epithelial cells and fibroblasts (37, 38). Human IL-33 genes are located on chromosome 9p24.1 and are composed of 270 amino acids, including N-terminal and C-terminal domains. Nuclear localization sequences and chromosome binding regions are located in the N-terminal structure (AA1-65); the IL1-like cytokine domain is included in the C-terminal domain (AA112-270) (39). Because of these properties of the protein structure, IL-33 has dual functions, acting both as a cytokine and as a transcriptional regulator. When facing danger or stress, IL33 is released from cells and binds to the ligand ST2 to exert an immune effect. IL33/ST2 promotes the phosphorylation and activation of PI3K/AKT, p38, ERK1/2, and JNK signalling, participating in both normal physiology and various diseases (40–42). In addition, IL33 in the nucleus may play a role in some physiological activity independently of ST2. However, recent studies have pointed out that nuclear IL33 has no function in gene transcription in endothelial or epithelial cells (43, 44). Initial studies illustrated that IL-33 acted as a transcriptional regulator to inhibit NF-KB transcriptional activity (45). Nuclear IL-33 in epidermal keratinocytes is involved in the pathogenesis of atopic dermatitis and serves as a cofactor of STAT3, inhibiting keratinocyte differentiation and skin barrier function *via* phosphorylation of STAT3 and STAT6 (46). In intervertebral disc degeneration, IL33 regulates ECM degradation independent of ST2 (47). Previous studies suggested that disordered IL33/ST2 signalling is involved in endometrial decidualization and embryo implantation, but the specific mechanism has not been elucidated. In the present study, we use Ishikawa cells, which express estrogen and progesterone receptors, as an endometrial epithelium model. We found the endogenous IL33 mRNA and protein levels were increased in Ishikawa cells treated with estrogen and progesterone in a time-dependent manner. In the mouse model, IL33 expression was strengthened at Dpc.4.5, which was the period of mouse embryo implantation. Correspondingly, the expression of IL33 in secretory phase endometrium is higher than that in proliferative phase in previous study (18). These studies indicated that intracellular IL33 may be regulated by estrogen and progesterone and involved in embryo implantation *in vivo and in vitro*. Besides, our results were consistent with previous findings that nuclear IL33 expression is upregulated during the secretory phase (the implantation window), suggesting the important role of nuclear IL33 in this process. In addition, when ST2 was silenced, the promoting effect of IL33 was not weakened, indicating that IL33 in the nucleus could still exert its corresponding function. There were some limitations to our study, which should be mentioned. Although we identified one possible mechanism of IL33, that is, the regulation of STAT3 phosphorylation, we did not further explore the transcriptional regulation of IL33 in the nucleus. This requires additional research in the future.

## Conclusion

In conclusion, the present study first demonstrated that nuclear IL33 plays an important role in epithelial cells by inducing HOXA10 expression *via* the phosphorylation of STAT3, which is critical for mouse and human pregnancy establishment and maintenance. This study provides a new pathological mechanism study for infertility in adenomyosis and may bring new insights for the treatment of infertility in adenomyosis. The specific molecular mechanism still needs to be further studied.

## Data Availability Statement

The raw data supporting the conclusions of this article will be made available by the authors, without undue reservation.

## Ethics Statement

The studies involving human participants were reviewed and approved by Ethics Committee of Shanghai First Maternity and Infant Health Hospital. The patients/participants provided their written informed consent to participate in this study. The animal study was reviewed and approved by Ethics Committee of Shanghai First Maternity and Infant Health Hospital.

## Author contributions

K-ML and QY contributed to the conception and design of the work. MZ, Z-QC contributed to the acquisition. BH, FH and X-MT drafted the manuscript. All authors contributed to the article and approved the submitted version.

## Funding

This work was supported by the National Natural Science Foundation of China [grant number 81901560 and 82102631].

## Conflict of interest

The authors declare that the research was conducted in the absence of any commercial or financial relationships that could be construed as a potential conflict of interest.

## Publisher’s note

All claims expressed in this article are solely those of the authors and do not necessarily represent those of their affiliated organizations, or those of the publisher, the editors and the reviewers. Any product that may be evaluated in this article, or claim that may be made by its manufacturer, is not guaranteed or endorsed by the publisher.
